# Hospitals and the Holiday Season

**Published:** 1915-05-29

**Authors:** 


### HOSPITALS AND THE HOLIDAY SEASON.

If men are to accomplish efficient work they must have occasional holidays. Such is the general creed and we do not propose to dispute it. But holidays this year have to be arranged and enjoyed under special conditions, and indeed full enjoyment and the setting aside of all anxieties is in the nature of the case hardly possible. Still there must be a break in the personal labours of many, even if health rather than happiness is the purpose to be attained. Members of the medical profession are taking an active share in the present national responsibilities and activities and the time is at hand when they must be framing their holiday plans. The difficulties this year will be great in view of the numbers called to the aid of the military services, and probably in not a few instances the holiday will have to be restricted even to more modest proportions than usual. *Locum tenentes* will be difficult to secure, and they are terribly expensive withal. In these circumstances there is a plain indication that the helpful principle of co-operation must be invoked. On the individual scale this has no doubt been the custom in the past, but in the present circumstances something wider and more definitely organised is needed. Examples have been set by a few districts, and particulars can easily be obtained by those who desire them. We are far from despising the spirit of comradeship and professional tradition which characterises these proposals, but we are at the same time glad to recognise their sensible and businesslike basis. Equipped with this quality they are likely to be permanent and to stand the stress of circumstance.

What is being attempted in private practice in the direction just indicated is worthy of the attention of our hospital staffs. Seeing how short-handed many of these are there will be special difficulty during the holiday season in keeping hospital work at a satisfactory level. Indeed, we do not see how this can be done except at the expense of a good deal of personal sacrifice. Organisation, however, can accomplish much, and especially if it is linked with foresight and is put into operation without delay. We know full well that many hospital physicians and surgeons have already far exceeded their statutory duties, and we recognise that they must have at least a measure of relief. Our desire is to urge that arrangements for this should be made in advance, and that these should be planned in such a fashion as to allow the hospital work to continue. Unless there is organisation to this end we fear that a conflict will arise between the convenience of the individual and the continuation of the service.

What ought to be urged most strongly is that further curtailment of hospital facilities will be a grievous injury to the poorer classes in our large towns. It is an easy solution of difficulties to close wards or to shut up out-patient departments. But such a course is hardly evidence of statesmanship, and we cannot think it justified except as a last resource. If it can be adopted by any institution without causing suffering and harm it becomes a fair question whether such an institution is not wholly superfluous and whether its closure might not well be a permanent one. We appreciate the difficulties both of the governing bodies and of the medical staffs, and we are sure that the special obligations of the hour press heavily upon them. But the time is one for sacrifice in the general interest, and here is an opportunity carrying a special sanction. Apart from the wider interest there is the appeal of what we may call local patriotism. Special honour will be due to those who in times of national stress and local anxieties address themselves successfully to meet the position in such a fashion that the help needed by their poorer neighbours is continued in full operation. Individuals must have rest and relaxation, but it is the special privilege of charity never to fail.

